# Impact of changes in antihypertensive medication on treatment intensity at hospital discharge and 30 days afterwards

**DOI:** 10.3389/fphar.2024.1376002

**Published:** 2024-08-09

**Authors:** Nuša Japelj, Mojca Kerec Kos, Maja Jošt, Lea Knez

**Affiliations:** ^1^ Faculty of Pharmacy, University of Ljubljana, Ljubljana, Slovenia; ^2^ Department of Pharmacy, University Clinic Golnik, Golnik, Slovenia

**Keywords:** hypertension, antihypertensive agents, hospitalization, patient discharge, continuity of patient care

## Abstract

**Introduction:**

Little is known about the cumulative effect of changes in antihypertensive medications on treatment intensity. This study analyzed how changes in antihypertensive medications affect the intensity of antihypertensive treatment at hospital discharge and 30 days afterwards.

**Methods:**

A prospective observational study of 299 hospitalized adult medical patients with antihypertensive therapy was conducted. The effect of medication changes on treatment intensity was evaluated by the Total Antihypertensive Therapeutic Intensity Score (TIS).

**Results:**

At discharge, antihypertensive medications were changed in 62% of patients (184/299), resulting in a very small median reduction in TIS of −0.16. Treatment intensity was reduced more with increasing number of antihypertensive medications at admission, whereas it increased with elevated inpatient systolic blood pressure. Thirty days after discharge, antihypertensive medications were changed in 37% of patients (88/239) resulting in a median change in TIS of −0.02. Among them, 90% (79/88) had already undergone a change at discharge. The change in treatment intensity after discharge was inversely correlated with a change at discharge.

**Discussion:**

Changes in antihypertensive medication frequently occurred at discharge but had a minimal impact on the intensity of antihypertensive treatment. However, these adjustments exposed patients to further medication changes after discharge, evidencing the need for treatment reassessment in the first month post-discharge.

## 1 Introduction

Hospitalization poses patients to a generalized risk for adverse events shortly after hospital discharge (Krumholz, 2013) and medication confusion during care transitions greatly contributes to this risk. At discharge, almost every hospitalized patient experiences changes in medications (Grimmsmann et al., 2007; Mansur et al., 2008; Unroe et al., 2010). Changes in antihypertensive medications are frequent (Himmel et al., 1996; Müller-Bühl et al., 2008; Viktil et al., 2012; Harris et al., 2013; Chung et al., 2022), and occur in nearly 40% of hospitalized patients with hypertension (Alhawassi et al., 2015). Hypertension is extremely common among inpatients, although it is usually not the primary reason for hospitalization. Therefore, the benefits of optimizing antihypertensive treatment during hospitalization should be carefully weighed against the risks associated with non-essential medication changes at hospital discharge (Anderson et al., 2018; Anderson et al., 2019).

Modifying antihypertensive therapy at hospital discharge is challenging due to the numerous factors that influence inpatient blood pressure. In studies where intensification of antihypertensive therapy at discharge was triggered solely by elevated inpatient blood pressure, blood pressure control did not improve and the risk of cardiovascular events within 1 year was not reduced (Anderson et al., 2018; Anderson et al., 2019; Rastogi et al., 2021). In addition, intensification of antihypertensive therapy at discharge was shown to increase patients’ risk for hospital readmission and serious adverse events (Butt et al., 2013; Shimbo et al., 2016; Anderson et al., 2019; Rastogi et al., 2021). Outside hypertension emergencies, current guidelines do not specifically address modifications in antihypertensive treatment in hospital settings (Williams et al., 2018). As recommendations on hypertension treatment in outpatient settings cannot be directly applied to inpatients, non-essential changes should be postponed until after hospitalization (Aung et al., 2015). Nevertheless, at discharge, changes in antihypertensive therapy may be necessary due to treatment of other conditions, such as decompensated heart failure, acute myocardial infarction and stroke. Although these changes are expected to affect blood pressure control, accurate assessment of the overall effect on the intensity of antihypertensive treatment is lacking. Existing studies have mostly focused on only one type of change, mainly intensification, and only one time point, usually hospital discharge.

Thus, the aim of this study was to analyze how changes in antihypertensive medications affect the intensity of antihypertensive treatment at hospital discharge and 30 days afterwards.

## 2 Materials and methods

### 2.1 Study design and ethical standards

A prospective observational study in 299 adult medical patients, hospitalized at the University Clinic Golnik, Slovenia, was conducted. The study was approved by the National Medical Ethics Committee of the Republic of Slovenia (Protocol Number 0120-223/2019/4). All procedures performed in the study were in accordance with the Declaration of Helsinki and comparable ethical standards. Informed consent was obtained from all included individuals.

### 2.2 Study population

**FIGURE 1:**
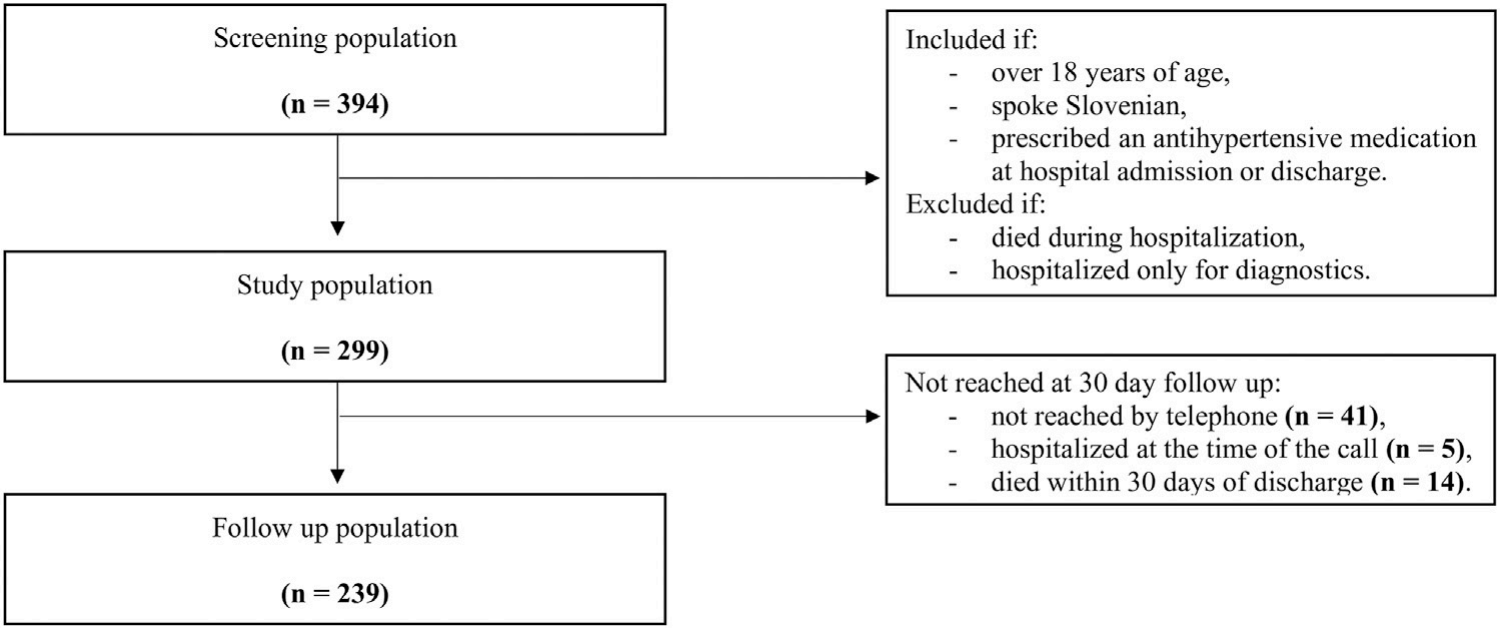
Flow diagram of the study population with inclusion and exclusion criteria. Patients were included between September 30, 2019, and October 15, 2020. The study was interrupted on March 12, 2020, because of epidemic proclamation in Slovenia. Inclusion was continued on August 26, 2020.

Patients were recruited gradually to guarantee feasibility of follow-up after discharge. On each working day, 15% of admitted patients were randomly selected from all hospitalized medical patients using the Research Randomizer (Research Randomizer, 2007). Patients who were over 18 years of age and spoke Slovenian were included in the screening population (Figure 1). Further on, patients who had been prescribed an antihypertensive medication at hospital admission or discharge were included in the study population. We aimed to include approximately 300 patients.

### 2.3 Data collection

Data collection was performed by researchers in a standardized fashion. Comprehensive medication history was obtained by interviewing patients after review of their medical documentation and electronic records of dispensed medications. Patients’ medical information and discharge therapy were obtained from hospital medical records. Patients were interviewed by telephone to obtain information on medication therapy 30 days after discharge.

Patients’ diagnoses were reviewed to determine the presence of acute cardiovascular condition at admission (e.g., unstable angina pectoris, acute myocardial infarction, uncontrolled blood pressure, decompensated heart failure, stroke, atrial fibrillation, ischemic heart disease). Patients without acute cardiovascular conditions were primarily hospitalized for other common reasons prevalent among medical patients at this clinic, such as infections, respiratory diseases, or malignancies. The blood pressure was measured during hospitalization by registered nurses according to hospital standards. Inpatient systolic blood pressure was categorized as severely (above 180 mmHg), moderately (above 160 mmHg), mildly (above 140 mmHg) or not elevated (below 140 mmHg) based on the three highest recordings during hospitalization. Comorbidity burden was calculated using the Charlson Comorbidity Index (Charlson et al., 1987).

### 2.4 Study outcomes

The outcomes were changes in antihypertensive medications and the intensity of antihypertensive treatment at and 30 days after discharge.

Changes in antihypertensive medications at hospital discharge were identified by comparing medication history at admission and medication lists at discharge, while changes 30 days after discharge were determined by comparing medication lists at discharge and medication therapy reported at 30 days after discharge.

To quantify the impact of medication changes on the intensity of antihypertensive treatment, the Total Antihypertensive Therapeutic Intensity Score (TIS) was calculated. TIS is determined by summing up the proportions of the prescribed doses relative to the maximum recommended doses for each antihypertensive medication a patient is taking. For example, if a study participant was prescribed two antihypertensive medications, each at 50% of the maximum recommended dose, TIS would be 1.0 (0.5 + 0.5). The maximum contribution of an individual medication to TIS was limited to the value 2 (corresponding to two maximum recommended doses) to avoid a substantial increase in TIS without a proportional effect on blood pressure, for example, when high doses of loop diuretics are used for oedema treatment (Koda-Kimble et al., 2005). The maximum daily dose was derived from the summary of product characteristics or the latest guidelines for the management of arterial hypertension (Williams et al., 2018). A one-point increase in TIS is expected to result in a 14–16 mmHg decrease in systolic blood pressure (Levy et al., 2016). For each patient, TIS was calculated at three time points: at admission, at discharge and 30 days after discharge. Further, TIS changes at discharge (difference between TIS at discharge and at admission) and TIS changes at 30 days after discharge (difference between TIS 30 days after discharge and at discharge) were calculated.

### 2.5 Statistical analysis

Data are presented using descriptive statistics. A logistic regression model was developed to analyze associations between patient or hospitalization characteristics and a change in antihypertensive medications. A linear regression model was developed to analyze associations between patient or hospitalization characteristics and TIS changes. In both regression analyses, univariable analysis was performed first followed by multivariable analysis. The regression coefficient reported for the logistic regression model was odds ratio (OR), and for the linear regression model, the value B. For linear regression models, diagnostic analysis of residuals was also performed. A *p*-value of less than 0.05 was considered statistically significant. All statistical analyses were performed using the statistical program IBM SPSS Statistics version 29.0.

## 3 Results

### 3.1 Characteristics of patients and antihypertensive therapy at hospital admission, discharge and 30 days afterwards

The study population consisted of 299 patients who had been prescribed an antihypertensive medication at hospital admission or discharge (Figure 1). The patients were mostly elderly, with a median age of 73 years, 55% were men (164/299), and the majority had multiple comorbidities, as evidenced by 46% (137/299) having a Charlson Comorbidity Index of three or more (Table 1). Patients were taking a median of eight medications at admission. An acute cardiovascular condition was identified in 35% of the patients (105/299), with decompensated heart failure occurring in 20% (61/299), atrial fibrillation in 4% (11/299), and uncontrolled blood pressure in 3% (8/299). During hospitalization, only 2% (5/299) and 12% (35/299) of patients had severely and moderately elevated blood pressure, respectively (Table 1).

**TABLE 1:** Characteristics of the included patients and antihypertensive therapy at hospital admission (n = 299), at hospital discharge (n = 299) and 30 days after discharge (n = 239).

Characteristic	Value
Age in years, median (IQR)	73 (66–81)
Male sex, n (%)	164 (55)
Charlson Comorbidity Index, n (%)	
0	26 (9)
1	71 (24)
2	65 (22)
≥3	137 (46)
Acute cardiovascular condition at admission, n (%)	
Decompensated heart failure	61 (20)
Atrial fibrillation	11 (4)
Uncontrolled blood pressure	8 (3)
Acute myocardial infarction	7 (2)
Unstable angina pectoris	6 (2)
Stroke	1 (0.3)
Ischemic heart disease	1 (0.3)
Other	10 (3)
Number of all medications at admission, median (IQR)	8 (6–11)
Length of hospitalization in days, median (IQR)	8 (5–11)
Inpatient systolic blood pressure, n (%)	
Severely elevated (≥180 mmHg)	5 (2)
Moderately elevated (160–179 mmHg)	35 (12)
Mildly elevated (140–159 mmHg)	95 (32)
Not elevated (<140 mmHg)	164 (55)
Number of antihypertensive medications, median (IQR)	
At admission	2 (1–3)
At discharge	2 (1–3)
30 days after discharge	2 (1–3)
Intensity of antihypertensive treatment, median (IQR)	
At admission	1.25 (0.56–2.25)
At discharge	1.06 (0.50–2.00)
30 days after discharge	1.06 (0.50–2.00)

IQR: interquartile range.

At admission, patients were using 702 antihypertensive medications in total, covering all the major drug classes for hypertension treatment (Supplementary Figure S1). The median of two antihypertensive medications was used per patient, yet the median TIS was only 1.25, suggesting that these medications were often not taken at the maximum recommended daily doses (Table 1). Antihypertensive therapy at hospital discharge was generally similar to that at admission, with patients prescribed a median of two antihypertensive medications and a median TIS of 1.06 (Table 1; Supplementary Figure S1). After hospital discharge, the antihypertensive therapy resembled the ones at admission and discharge, with patients taking a median of two antihypertensive medications and a median TIS of 1.06 (Table 1; Supplementary Figure S1).

### 3.2 Changes in antihypertensive therapy at hospital discharge

At discharge, 62% of patients (184/299) had at least one antihypertensive medication changed. Patients with a change in antihypertensive medications experienced a median of two changes per patient (IQR, 1–2). They were more often male (adjusted OR = 0.39, *p* = 0.001), prescribed a higher number of antihypertensive medications at admission (adjusted OR = 1.37, *p* = 0.005), had an acute cardiovascular condition (adjusted OR = 3.92, *p* < 0.001) and longer hospitalization (adjusted OR = 1.10, *p* = 0.005; Table 2).

**TABLE 2:** Factors associated with a change in antihypertensive medications at hospital discharge in the univariable and multivariable regression models (n = 299). A change in antihypertensive medications was considered to be any of the following: drug discontinuation or initiation, dose increase or decrease, and drug substitution within drug class.

Characteristic	Without a change^a^ (n = 115)	With a change^a^ (n = 184)	Univariable model		Multivariable model^b^	
			Unadjusted OR (95% CI)	p-value^c^	Adjusted OR (95% CI)	p-value^c^
Age in years	72 (64–79)	73 (67–82)	1.03 (1.00–1.05)	**p = 0.028**	1.02 (0.997–1.05)	p = 0.081
Gender				**p = 0.002**		**p = 0.001**
Male	50 (43)	114 (62)	Reference		Reference	
Female	65 (57)	70 (38)	0.47 (0.29–0.76)		0.39 (0.23–0.69)	
Charlson Comorbidity Index						
0	10 (9)	16 (9)	Reference		Reference	
1	36 (31)	35 (19)	1.10 (0.63–1.92)	p = 0.287	0.63 (0.23–1.74)	p = 0.372
2	22 (19)	43 (23)	1.22 (0.48–3.14)	p = 0.677	0.90 (0.31–2.61)	p = 0.841
≥3	47 (41)	90 (49)	1.12 (0.50–2.84)	p = 0.684	0.73 (0.27–1.98)	p = 0.532
Acute cardiovascular condition at admission				**p < 0.001**		**p < 0.001**
No	96 (83)	98 (53)	Reference		Reference	
Yes	19 (17)	86 (47)	4.43 (2.51–7.85)		3.92 (2.12–7.25)	
Number of antihypertensive medications at admission	2 (1–3)	3 (2–4)	1.28 (1.06–1.54)	**p = 0.011**	1.37 (1.10–1.72)	**p = 0.005**
Length of hospitalization in days	7 (5–9)	8.5 (6–12)	1.10 (1.04–1.16)	**p = 0.001**	1.10 (1.03–1.17)	**p = 0.005**
Elevated (>140 mmHg) inpatient systolic blood pressure				p = 0.485		p = 0.743
No	66 (57)	98 (53)	Reference		Reference	
Yes	49 (43)	86 (47)	1.18 (0.74–1.89)		1.10 (0.63–1.92)	

^a^ Characteristics of patients with or without a change in antihypertensive medications at discharge are also reported. Results are given as median values (with the interquartile ranges) or as number (with percentage) of patients.

^b^ Logistic regression model adjusted for all listed factors; Omnibus test: p < 0.001; Nagelkerke *R* ^2^ = 0.260; Constant = e^−2.380^.

^c^ Factors significantly associated with a change in antihypertensive medications are highlighted in bold.

**FIGURE 2:**
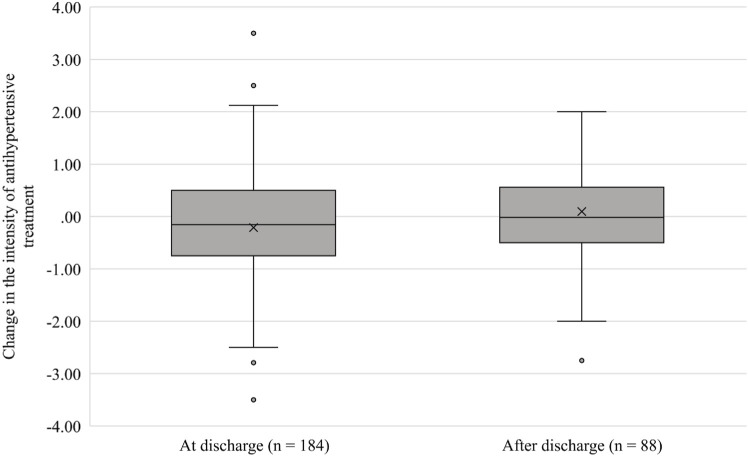
Boxplot of changes in the intensity of antihypertensive treatment in patients with a change in antihypertensive medication at hospital discharge and 30 days after discharge. A change in the intensity of antihypertensive treatment was calculated as the difference between the TIS value at discharge and at admission/ after discharge and at discharge. The median, mean, first and third quartiles (Q3–Q1) are shown.

In patients with a change in antihypertensive medications at discharge, the median change in TIS was just −0.16 (IQR, −0.75 to 0.50; Figure 2) indicating an expected increase in systolic blood pressure of less than 5 mmHg (Levy et al., 2016). There was a more pronounced decrease in treatment intensity among patients with more antihypertensive medications at admission (adjusted B = −0.328, *p* < 0.001; Table 3). Conversely, an increase in treatment intensity was more common among patients who had elevated inpatient systolic blood pressure (adjusted B = 0.283, *p* = 0.042).

**TABLE 3:** Factors associated with changes in the intensity of antihypertensive treatment at hospital discharge in the univariable and multivariable regression models (n = 184). A change in the intensity of antihypertensive treatment at discharge was calculated as the difference between the TIS value at discharge and at admission. A positive regression coefficient B represents a positive change in TIS at discharge, i.e., an increase in the intensity of antihypertensive therapy at discharge.

Characteristic	Univariable model		Multivariable model^a^	
	Unadjusted B (95% CI)	p-value^*b*^	Adjusted B (95% CI)	p-value^*b*^
Age in years	−0.13 (−0.026; 3.7*10^−4^)	p = 0.057	−0.010 (−0.022; 0.002)	p = 0.099
Gender		p = 0.227		p = 0.892
Male	Reference		Reference	
Female	−0.185 (−0.486; 0.116)		−0.019 (−0.292; 0.254)	
Charlson Comorbidity Index				
0	Reference		Reference	
1	0.307 (−0.294; 0.909)	p = 0.314	0.470 (−0.070; 1.011)	p = 0.088
2	0.023 (−0.561; 0.606)	p = 0.939	0.234 (−0.290; 0.758)	p = 0.380
≥3	0.114 (−0.427; 0.654)	p = 0.679	0.404 (−0.084; 0.892)	p = 0.104
Acute cardiovascular condition at admission		p = 0.362		p = 0.097
No	Reference		Reference	
Yes	0.136 (−0.158; 0.430)		0.220 (−0.040; 0.480)	
Number of antihypertensive medications at admission	−0.334 (−0.423; −0.245)	**p < 0.001**	−0.328 (−0.420; −0.237)	**p < 0.001**
Length of hospitalization in days	0.009 (−0.011; 0.029)	p = 0.376	−0.002 (−0.020; 0.016)	p = 0.834
Elevated (>140 mmHg) inpatient systolic blood pressure		p = 0.053		**p = 0.042**
No	Reference		Reference	
Yes	0.287 (−0.004; 0.578)		0.283 (0.011; 0.554)	

TIS: total antihypertensive therapeutic intensity score.

^a^ Linear regression model adjusted for all listed factors; adjusted *R* ^2^ = 0.244; Constant = 0.809.

^b^ Factors significantly associated with a change in antihypertensive medications are highlighted in bold.

Changes were made in 44% of antihypertensive medications (352/804). The most common change was drug discontinuation (128/804; 16%), followed by drug initiation (102/804; 13%) and dose increase (53/804; 7%) or decrease (58/804; 7%; Table 4). The changes most frequently involved loop diuretics (81/124; 65%), mainly as drug initiation, dose increase or decrease.

**TABLE 4:** Changes in antihypertensive medications at hospital discharge.

Class of antihypertensive medication	Total number of medications at admission and discharge	Any change	Drug discontinuation	Drug initiation	Dose increase	Dose decrease	Drug substitution within drug class
	n (%)	n (%)					
**All**	**804 (100)**	**352 (44)**	**128 (16)**	**102 (13)**	**53 (7)**	**58 (7)**	**11 (1)**
RAAS medications	234 (100)	97 (41)	47 (20)	20 (9)	10 (4)	18 (8)	2 (1)
Beta blockers	174 (100)	62 (36)	5 (3)	27 (16)	15 (9)	9 (5)	6 (3)
Calcium channel blockers	122 (100)	47 (39)	27 (22)	8 (7)	7 (6)	5 (4)	—
Thiazides	97 (100)	41 (42)	34 (35)	7 (7)	—	—	—
Loop diuretics	124 (100)	81 (65)	4 (3)	32 (26)	18 (15)	24 (19)	3 (2)
Aldosterone receptor antagonists	30 (100)	13 (43)	5 (17)	7 (23)	—	1 (3)	—
Other^a^	23 (100)	11 (48)	6 (26)	1 (4)	3 (13)	1 (4)	—

RAAS: angiotensin-converting enzyme inhibitors and angiotensin-II receptor blockers; thiazides: thiazide and thiazide-like diuretics. The bold values represent the total number (and percentage) of medications at admission and discharge, as well as the total number (and percentage) of all changes, categorized by the type of change.

^a^ Centrally acting antihypertensive medications (n = 22) and α-adrenoceptor antagonists (n = 1).

### 3.3 Changes in antihypertensive therapy 30 days after hospital discharge

After discharge, antihypertensive medications were changed in 37% of patients (88/239). Patients with a change in antihypertensive medications experienced a median of one change per patient (IQR, 1–2). Changes occurred more frequently in patients whose antihypertensive therapy had already been changed at discharge (adjusted OR = 13.66, *p* < 0.001; Table 5). In fact, in 90% of patients (79/88) who had a change in antihypertensive medications 30 days after discharge, the therapy had already been changed at discharge.

**TABLE 5:** Factors associated with a change in antihypertensive medications 30 days after hospital discharge in the univariable and multivariable regression models (n = 239). A change in antihypertensive medications was considered to be any of the following: drug discontinuation, drug initiation, dose increase, dose decrease, and drug substitution within drug class.

Characteristic	Without a change^a^ (n = 151)	With a change^a^ (n = 88)	Univariable model		Multivariable model^b^	
			Unadjusted OR (95% CI)	p-value *^c^*	Adjusted OR (95% CI)	p-value *^c^*
Age in years	72 (65–80)	73 (66–81)	1.00 (0.98–1.03)	p = 0.982	0.99 (0.96–1.02)	p = 0.525
Gender				p = 0.069		p = 0.787
Male	76 (50)	55 (63)	Reference		Reference	
Female	75 (50)	33 (38)	0.61 (0.36–1.04)		0.92 (0.48–1.74)	
Charlson Comorbidity Index						
0	11 (7)	12 (14)	Reference		Reference	
1	41 (27)	17 (19)	0.38 (0.14–1.03)	p = 0.057	0.49 (0.14–1.66)	p = 0.250
2	33 (22)	21 (24)	0.58 (0.22–1.56)	p = 0.283	0.50 (0.15–1.64)	p = 0.251
≥3	66 (44)	38 (43)	0.53 (0.21–1.31)	p = 0.196	0.54 (0.17–1.69)	p = 0.287
Acute cardiovascular condition				**p = 0.019**		p = 0.881
No	110 (73)	51 (58)	Reference		Reference	
Yes	41 (27)	37 (42)	1.95 (1.12–3.39)		0.95 (0.49–1.84)	
Number of antihypertensive medications at discharge	2 (1–3)	2 (1–3)	1.05 (0.84–1.31)	p = 0.663	1.00 (0.77–1.29)	p = 0.970
A change in antihypertensive medications at discharge				**p < 0.001**		**p < 0.001**
No	91 (60)	9 (10)	Reference		Reference	
Yes	60 (40)	79 (90)	13.31 (6.21–28.54)		13.66 (6.08–30.71)	

^a^ Characteristics of patients with or without a change in antihypertensive medications 30 days after discharge are also reported. Results are given as median values (with the interquartile ranges) or as number (with percentage) of patients.

^b^ Logistic regression model adjusted for all listed factors; Omnibus test: p < 0.001; Nagelkerke *R* ^2^ = 0.332; Constant = e^−0.985^.

^c^ Factors significantly associated with a change in antihypertensive medications are highlighted in bold.

In patients with a change in antihypertensive medications after discharge, alteration in treatment intensity was negligible, with a median change in TIS of −0.02 (IQR, −0.50 to 0.56; Figure 2). Importantly, the change in treatment intensity after discharge was inversely correlated with a change in treatment intensity at discharge (adjusted B = −0.401, *p* < 0.001; Table 6). There was a more pronounced decrease in treatment intensity among patients with more antihypertensive medications at discharge (adjusted B = −0.137, *p* = 0.031).

**TABLE 6:** Factors associated with changes in the intensity of antihypertensive treatment 30 days after hospital discharge in the univariable and multivariable regression models (n = 88). A change in the intensity of antihypertensive treatment after discharge was calculated as the difference between the TIS value after discharge and at discharge. A positive regression coefficient B represents a positive change in TIS after discharge, i.e., an increase in the intensity of antihypertensive therapy after discharge.

Characteristic	Univariable model		Multivariable model^a^	
	Unadjusted B (95% CI)	p-value^b^	Adjusted B (95% CI)	p-value^*b*^
Age in years	0.008 (−0.006; 0.022)	p = 0.246	0.009 (−0.004; 0.022)	p = 0.164
Gender		p = 0.761		p = 0.612
Male	Reference		Reference	
Female	0.056 (−0.311; 0.424)		−0.082 (−0.405; 0.240)	
Charlson Comorbidity Index				
0	Reference		Reference	
1	−0.492 (−1.099; 0.116)	p = 0.111	−0.281 (−0.841; 0.280)	p = 0.322
2	−0.244 (−0.828; 0.339)	p = 0.407	−0.190 (−0.716; 0.337)	p = 0.475
≥3	−0.691 (−1.225; −0.158)	**p = 0.012**	−0.454 (−0.965; −0.057)	p = 0.081
Acute cardiovascular condition at admission		p = 0.233		p = 0.240
No	Reference		Reference	
Yes	0.216 (−0.142; 0.574)		0.194 (−0.132; 0.520)	
Number of antihypertensive medications at discharge	−0.167 (−0.296; −0.038)	**p = 0.012**	−0.137 (−0.261; −0.013)	**p = 0.031**
TIS change at discharge	−0.476 (−0.651; −0.301)	**p < 0.001**	−0.401 (−0.579; −0.222)	**p < 0.001**

TIS: total antihypertensive therapeutic intensity score.

^a^ Linear regression model adjusted for all listed factors; adjusted R^2^ = 0.294; Constant = –0.078.

^b^ Factors significantly associated with a change in antihypertensive medications are highlighted in bold.

Changes affected 22% of antihypertensive medications (126/574). The most common change was drug initiation (43/574; 7%), followed by dose decrease (33/574; 6%), discontinuation of the drug (27/574; 5%) and dose increase (21/574; 4%; Table 7).

**TABLE 7:** Changes in antihypertensive medications 30 days after hospital discharge.

Class of antihypertensive medication	Total number of medications at discharge and after discharge	Any change	Drug discontinuation	Drug initiation	Dose increase	Dose decrease	Drug substitution within drug class
	n (%)						
**All**	**574 (100)**	**126 (22)**	**27 (5)**	**43 (7)**	**21 (4)**	**33 (6)**	**2 (0.3)**
RAAS medications	170 (100)	32 (19)	9 (5)	14 (8)	3 (2)	6 (4)	—
Beta blockers	131 (100)	18 (14)	3 (2)	1 (1)	4 (3)	9 (7)	1 (1)
Calcium channel blockers	86 (100)	26 (30)	6 (7)	11 (13)	4 (5)	4 (5)	1 (1)
Thiazides	57 (100)	9 (16)	2 (4)	7 (12)	—	—	—
Loop diuretics	92 (100)	33 (36)	6 (7)	5 (5)	10 (11)	12 (13)	—
Aldosterone receptor antagonists	22 (100)	2 (9)	1 (5)	1 (5)	—	—	—
Other^a^	16 (100)	6 (38)	—	4 (25)	—	2 (13)	—

RAAS: angiotensin-converting enzyme inhibitors and angiotensin-II receptor blockers; thiazides: thiazide and thiazide-like diuretics. The bold values represent the total number (and percentage) of medications at discharge and after discharge, as well as the total number (and percentage) of all changes, categorized by the type of change.

^a^ Centrally acting antihypertensive medications (n = 22) and α-adrenoceptor antagonists (n = 1).

## 4 Discussion

In this observational study of 299 hospitalized adult medical patients, changes in antihypertensive medications frequently occurred at hospital discharge, namely, in six out of 10 patients, but resulted in minimal impact on treatment intensity. After discharge, changes in antihypertensive medications occurred almost exclusively in patients whose therapy had been changed previously. Patients who had an increase in treatment intensity at discharge often experienced a decrease in treatment intensity after 30 days, and *vice versa*.

### 4.1 Changes in antihypertensive therapy at hospital discharge

In our cohort of patients, who were elderly, had many comorbidities and were treated with multiple medications, changes in antihypertensive therapy at discharge were expected (Himmel et al., 1996; Grimmsmann et al., 2007; Mansur et al., 2008; Müller-Bühl et al., 2008; Unroe et al., 2010; Viktil et al., 2012; Harris et al., 2013; Alhawassi et al., 2015; Chung et al., 2022). In fact, at discharge, changes occurred in 62% of patients (184/299) and in 44% of antihypertensive medications (352/804), with some patients experiencing multiple changes. Interestingly, these changes did not translate into an appreciable effect on blood pressure. The observed median change in treatment intensity (TIS change: −0.16) is expected to result in an increase in systolic blood pressure of less than 5 mmHg (Levy et al., 2016). Many changes were driven by an initiation or dose change of loop diuretics and were coupled with alterations in other drug classes, usually prioritized for antihypertensive treatment, such as RAAS medications, calcium channel blockers and thiazides. Moreover, changes were more common in patients with an acute cardiovascular condition, suggesting that changes were most likely due to treatment of the acute cause for hospitalization rather than inpatient blood pressure management. These changes were sometimes followed by adjustments of antihypertensive therapy to prevent overtreatment of blood pressure, especially when inpatient systolic blood pressure was below 140 mmHg.

The previous studies warned against premature intensification of antihypertensive therapy at hospital discharge. These studies reported intensification in antihypertensive therapy in 9%–14% of discharged patients (Anderson et al., 2018; Rastogi et al., 2021). In the present study, changes were not associated with elevated inpatient systolic or diastolic blood pressure, but were more often observed in patients with an acute cardiovascular condition, consistent with findings from other studies (Anderson et al., 2018; Chung et al., 2022). Indeed, chronic disease markers such as inpatient blood pressure may vary due to acute reasons for hospitalization and other conditions such as acute pain, anxiety or additional medications. The small reduction in treatment intensity at hospital discharge observed in the current study is consistent with the recommendations of previous authors (Aung et al., 2015; Anderson et al., 2018; Rastogi et al., 2021) and recent antihypertensive-deprescribing initiatives in old (Reeve et al., 2020) and very old (Sheppard et al., 2020) individuals. Nevertheless, in the current study, numerous changes in antihypertensive medications were performed at hospital discharge. At discharge, any change in drug therapy should be carefully considered in light of the broader context of the individual patient, their drug therapy, and chronic and acute health status, not to increase the risk of adverse events (Butt et al., 2013; Shimbo et al., 2016; Anderson et al., 2019; Rastogi et al., 2021). If antihypertensive intensification is necessary, assessment of fall risk and implementation of fall prevention measures should be performed (Guideline for the Prevention of Falls in Older Persons, 2001; Tinetti and Kumar, 2010).

### 4.2 Changes in antihypertensive therapy 30 days after hospital discharge

After discharge, changes in antihypertensive medications occurred less frequently and were detected in 37% of patients (88/239) and in 22% of antihypertensive medications (126/574). Importantly, these changes were more often in patients whose antihypertensive medications had already been changed at discharge, and the intensity of antihypertensive treatment was inversely correlated between the two time points.

Changes in drug therapy shortly after hospital discharge were expected (Mansur et al., 2008; Müller-Bühl et al., 2008; Viktil et al., 2012). Most importantly, our study confirmed that treatment changes at discharge, even if constrained to antihypertensive medications as in our study, expose patients to further changes after discharge, possibly contributing to post-hospital syndrome (Krumholz, 2013). After discharge, patients’ therapy often needs to be readjusted due to improvements in their health or new health needs. In addition, drug therapy after hospitalization all too often requires reconciliation of unintentional discrepancies that arise from poor communication at transitions of care. Medication reconciliation should be implemented to assure changes in therapy are done purposely, documented clearly in discharge letters, and explained to patients to avoid medication confusion and to ensure patient safety (Unroe et al., 2010; Krumholz, 2013). Moreover, our results suggest the need for treatment evaluation within the first month after patients’ discharge.

### 4.3 Study strengths and limitations

The strength of this study is that it not only tracks changes in antihypertensive medications, but also assesses their overall impact on treatment intensity by using TIS for a comprehensive analysis. Additionally, these effects were assessed both at discharge and 30 days post-discharge, providing a deeper understanding of the impact of antihypertensive medication adjustments.

A limitation of the study was the lack of distinction between intentional and unintentional changes in antihypertensive therapy, which would provide additional insight into the relation between changes at discharge and afterwards. The actual effect of changes on blood pressure control was not measured, but their expected indirect effect was estimated with TIS that assumes equal response of each patient. No data on blood pressure were collected after discharge because blood pressure could not be measured in the same way as in the hospital. Only 394 of all 3,087 medical patients hospitalized during the 7-month study period were randomly selected for screening. Indeed, patients had to be recruited gradually to guarantee telephone interview after discharge. Inclusion of consecutive patients would avoid selection bias, however this was minimized by random selection of patients.

## 5 Conclusion

Six out of ten patients had changes in their antihypertensive medications at hospital discharge, predominantly those with an acute cardiovascular condition at admission. Changes in antihypertensive medications at discharge had minimal impact on treatment intensity, resulting in an expected systolic blood pressure increase of less than 5 mmHg. Thirty days after discharge, almost four out of ten patients experienced further changes in antihypertensive therapy, particularly those whose medications had already been altered at discharge. These results highlight the dynamics of antihypertensive therapy management in elderly patients with multiple comorbidities, emphasizing the need for careful consideration and follow-up in first month after discharge to optimize treatment and patient safety.

## Data Availability

The raw data supporting the conclusions of this article will be made avaliable by the authors on reasonable request. Access may be granted with appropriate permissions, ensuring participant anonymity and privacy are protected.
