# Supplementary figures and images for: Impact of changes in antihypertensive medication on treatment intensity at hospital discharge and 30 days afterwards
Source: Front Pharmacol. 2024 Aug 9;15:1376002. doi: 10.3389/fphar.2024.1376002 (PMC11341450; doi:10.3389/fphar.2024.1376002)

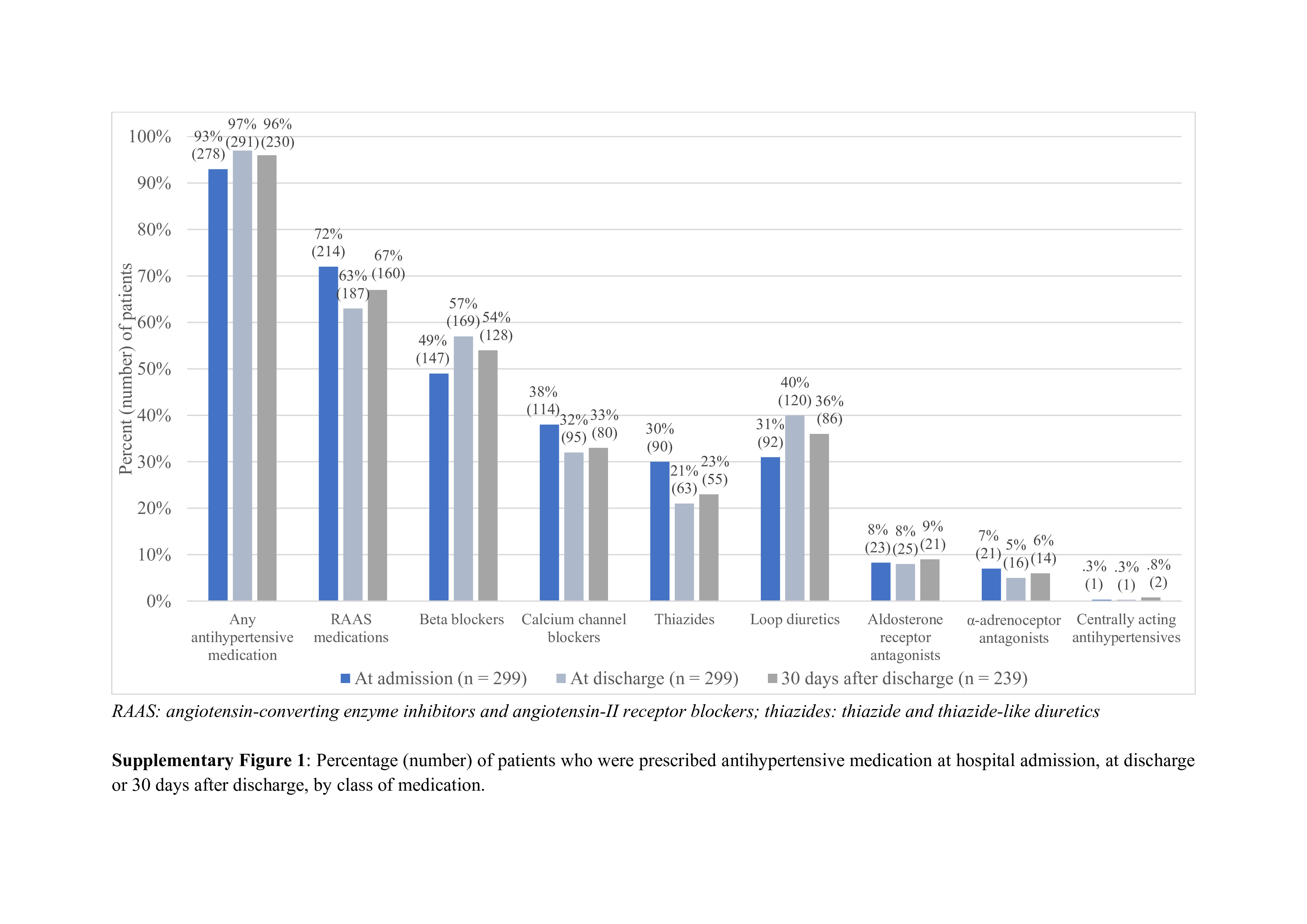

Supplement: Supplementary file 1 [file Image1.JPEG]
